# Mental health, duration of unemployment, and coping strategy: a cross-sectional study of unemployed migrant workers in eastern china during the economic crisis

**DOI:** 10.1186/1471-2458-12-597

**Published:** 2012-08-02

**Authors:** Li Chen, Wenhu Li, Jincai He, Lanhua Wu, Zheng Yan, Wenjie Tang

**Affiliations:** 1 Department of Psychology, School of Environmental Science and Public Health, Wenzhou Medical College, Wenzhou, China; 2 School of Psychology, Jiangxi Normal University, Nanchang, China; 3 Department of Educational and Counseling Psychology, School of Education, The University at Albany, State University of New York, Albany, New York, USA

**Keywords:** Mental health, Duration of unemployment, Coping strategy, Unemployed migrant workers, Economic crisis

## Abstract

**Background:**

20 million migrant workers in China lost their jobs during the economic crisis of 2008. Both urban migration and unemployment have long been documented to be associated with vulnerability to mental problems. This study aims to examine the mental health of unemployed migrant workers in Eastern China and its relation to duration of unemployment and coping strategy during the recent economic crisis.

**Methods:**

The data were collected through interview-based survey with a sample of 210 unemployed migrant workers in Zhejiang Province of China from 2008 to 2009. Symptom Checklist-90-Revised, Coping Strategies Questionnaire, and seven short demographic questions were used.

**Results:**

The majority of the unemployed migrant workers were found to be young male manufacturing industry workers with short-term unemployment and a relatively low education level. Nearly 50% of unemployed migrant workers were classified as mentally unhealthy and the most frequently reported symptom was depression. Compared with the adult norm of 1986, 2003, and 2007 in China, unemployed migrants had more mental problems. Long-term unemployed migrant workers had more psychiatric symptoms than the short-term unemployed workers and employed migrant workers. Unemployed migrant workers with immature coping strategies expressed significantly more psychiatric symptoms than those with mixed and mature coping strategies. Duration of unemployment and two coping strategies, problem-solving and self-blaming, predicted the mental problems of unemployed migrant workers.

**Conclusions:**

The results indicated that mental health status of unemployed migrant workers in Eastern China was poorer than the national adult norm. More psychiatric symptoms are evidenced among unemployed migrant workers who lost their jobs for a long term and who had immature coping strategies. These findings can be used for prevention and intervention of mental illness among unemployed migrant workers.

## Background

The worldwide economic crisis of 2008 resulted in massive unemployment across the world. In June 2009, for example, approximately 45 million (8.3%) people living in the member countries of Organisation for Economic Co-operation and Development (OECD) were unemployed and estimated unemployment rates in underdeveloped countries were even as high as 80-90% [1].

Unemployed people generally experience three major types of distress. The first is financial distress. They have to face financial ruin, and many begin to lose their lifetime savings and even their homes [2]. The second is physical health. Medical symptoms such as diabetes, hypertension, and coronary heart disease are associated with unemployment [3-5]. The third is mental health. Poor mental health is common among the unemployed [6,7] and unemployment is associated with higher levels of depression [8,9], suicide [10] and anxiety [11].

Published studies on the relationship between unemployment and mental health have extensively focused on developed countries in the West [7-10] and unemployed urban residences with labor insurance (e.g. nurses, white-collar employees, and stable workers) [12-14]. These studies not only have significantly advanced current knowledge concerning the mental health of unemployed urban individuals in the Western countries but also have motivated new important research questions. For example, what is the general relationship between unemployment and mental health during the economic crisis in East Asian countries such as China? What is the specific relationship between unemployment and mental health during the economic crisis among migrant workers as the typical urban workforce in China, especially those who are unemployed and without unemployment insurance?

The present study seeks to go beyond existing studies to further examine mental health among unemployed migrant workers during the economic crisis by making the following four specific efforts.

First, we explore mental health in the Chinese context. Either economic boom [15] or crisis [16-19] can generate mental problems. Many Chinese people have suffered double distress from both rapid economic growth and an unprecedented economic crisis in the recent years. Lee’s research group, for instance, found that economic contraction triggered by a global economic crisis of 2008 was associated with a higher risk of depression [20].

Second, we have chosen unemployed migrant workers as our subjects. In China, there are around 120 million rural–urban migrants, accounting for about 25% of the working population of the entire country [21]. The economic crisis of 2008 has forced thousands of labour-intensive enterprises to go bankrupt in China and hundreds of thousands of migrant workers to lose their jobs. According to China’s National Statistics Bureau, by the end of 2008, the registered unemployed population in urban areas was 8.86 million, and about 20 million of 120 million peasants who went to cities for work in China have lost their jobs [22]. In recent years, the mental health of migrant workers in China has received close attention by researchers [21,23-25]. For example, in an important study, Wong and his collaborators evaluated the mental health status of 475 migrant workers in Shanghai by using the Brief Symptoms Inventory (BSI). They found that migrant workers experienced more stress in “interpersonal tensions and conflicts” and were more likely to be mentally unhealthy [23].

Third, we focus on two aspects of unemployed workers, their duration of unemployment and coping strategies used. First, duration of unemployment refers to the time period during which the person recorded as unemployed was seeking or available for work. The positive linear relationship between duration of unemployment and mental health has been well studied in a number of populations [26-29]. Stankunas’ group, for instance, surveyed 429 unemployed persons in Lithuania and found that long-term unemployed persons had more episodes of depression in the past 12 months in comparison with short-term unemployed ones [26]. Second, coping strategies, according to the vulnerability-stress model proposed by Zubin and Spring [30], mediate the potentially negative effects of daily stressors and thus influence mental health [31,32]. The development of a successful coping behavior is likely to reduce stress and help a person to solve personal problems and maintain psychological balance and health [33]. People with a lower level of mental health have been shown to use more avoidance or other negative coping strategies, whereas people with a higher level of mental health use more problem-focused strategies aimed at dealing with the stressors themselves [32]. As one of the earliest empirical efforts, Li and his collaborators found that migrant workers were more inclined to turn to friends than family members and only in 1% of the cases sought professional help [21]. Thus, it is theoretically and practically important to examine duration of unemployment as a stressor and coping strategies as stress management skills in order to understand how unemployed migrant worker in China develop and deal with their mental problems.

The aims of the present paper were to (1) examine the frequency and severity of mental health problems among the unemployed migrant workers in Eastern China during the economic crisis and (2) evaluate the association of their mental health with unemployment duration and coping strategies.

## Methods

### Research design

The cross-sectional design was used in the study. The longitudinal design has widely been used to observe the changes of mental health over long periods of time [20,34]. However, it is a unique challenge to use the longitudinal design to study unemployed migrant workers in China. It is simply infeasible in China to track unemployed migrant workers because they change their jobs frequently and no formal mechanisms such as social welfare registrars in China exist to find them, except for identifying them individually in the non-organized job seeking market. On the other hand, it is important to study mental health of unemployed migrant workers during, rather than after, an economic crisis. Moreover, cross-sectional studies allow one to compare the mental health of different types of unemployed groups at a single time point (e.g., the beginning of economic crisis).

Interview-based survey has frequently been used to study mental health issues [35-37] and was chosen in the study for two specific reasons. First, unemployed migrant workers in China do not need to have labor insurance in order to register in the local labor market. Unlike in many other countries where the majority of unemployed individuals register at governmental or municipally-run agencies for receiving unemployment compensation, unemployment insurance is usually provided for the urban dwellers in China. It is impossible to use the telephone-based interview because of the lack of any information on individual unemployed migrant workers (such as telephone numbers). Thus, the best way for us to find interviewees is to concentrate on the labour markets and wait for these migrant job hunters. Second, many of unemployed migrant workers in China do not understand the meaning of psychological questionnaires due to their semi literacy or illiteracy.

### Participants

Migrant workers were defined in the study as those who were 18 years of age or older, possess a legal rural *hukou* (“户口”, formally registered permanent residents in a rural area in China), and have been granted the legal right to work temporarily in urban and prosperous coastal regions for at least six months. Unemployed workers, as defined by the International Labour Organization, refers to people who are without jobs and have been actively looking for work within the past four weeks [38]. These criteria were used in the study to identify participants.

We use the following method [39] to determine the sample size: Sample Size = n / [1 + (n/population)] while n = Z * Z [P (1-P)/ (D*D)]. Here, Z = 1.960 with Confidence Level of 95%; P (Expected Frequency Value) = 43.6%, based on our pilot study that the positive symptom detection rate of SCL-90 in the unemployed migrant workers is 43.6%); D (Maximum difference between the sample mean and the population mean) = 0.15P = 0.0654. Thus, the estimated sample size = 220. A total of 232 unemployed migrant workers were identified by convenience sampling for the interview-based survey. Among them, 22 individuals (9%) declined to participate and 210 individuals (91%) filled out all the questionnaires, which was the final sample size of the study.

### Measurement

#### Socio-demographics

A demographic questionnaire elicited basic background information, including age, gender, marital status, education, region of origin, household register, type of last job and other basic demographic information.

#### Duration of unemployment

The duration of unemployment was assessed by asking for the number of months unemployed altogether. Short-term unemployment is defined in this study as less than three months; long-term is greater than three months [38].

#### Mental health

Mental health status was measured by the Chinese version [40] of the Symptom Checklist-90-Revised (SCL-90-R) [41]. It is the most widely used instrument in China to examine mental disorders. From 1986 to 2007, there were over 500 independent studies used by SCL-90-R in China [42]. The SCL-90-R is a 90-item self-report symptom inventory designed to screen for a broad range of psychological problems. Each of the 90 items is rated on a five-point scale of distress, ranging from “not at all” (1) to “extremely” (5). The answers are combined in nine primary symptom dimensions: Somatization(SOM), Obsessive-Compulsive(O-C), Interpersonal Sensitivity(I-S), Hostility(HOS), Depression(DEP), Anxiety(ANX), Paranoid Ideation(PAR), Phobic Anxiety(PHOB) and Psychoticism(PSY). The internal consistency coefficient alphas for the nine symptom dimensions in the study ranged from 0.75-0.88. In addition, the severity of psychiatric and psychosomatic symptoms was defined by the score of the global severity index (GSI) [40]. Individual respondents who had GSI total scores greater than 70 points were considered to have poor mental health.

#### Coping strategy

Coping strategies were measured using an adapted Coping Strategies Questionnaire (CSQ) by Xiao and Xu [43], which was a revised version of a questionnaire developed by Folkman and Lazarus [44]. It contains 62 items to assess six factors, problem-solving, self-blaming, help-seeking, fantasizing, avoidance, and rationalization. These six factors comprise three types of coping strategies, immature type (self-blaming, fantasizing, and avoidance), mixed type (rationalization), and mature type (problem-solving, help-seeking). Internal consistency coefficients alphas obtained in the study were 0.80 for problem-solving, 0.82 for self-blaming, 0.85 for help-seeking, 0.81 for fantasizing, 0.80 for avoidance, and 0.90 for rationalization.

### Procedure

During the period from December 2008 to April 2009, unemployed migrant workers were identified and recruited in the recruitment hall of the buildings of two largest labor markets in two cities, Wenzhou and Ningbo, Zhejiang Provience, China. These two cities in Zhejiang Province were chosen as the study site because Zhejiang was the one of most seriously hit areas in China during the economic crisis of 2008; 20% of small and medium enterprises of Zhejiang was closed down, especially in Wenzhou and Ningbo.

The present study consisted of the following steps. First, in the pilot study, the questionnaire was pre-tested with 30 unemployed migrant workers from the labor market in the city of Wenzhou. Eight participants were unable to finish the questionnaire independently and found that many of them simply did not understand the meaning of psychological questionnaires due to their semi literacy or illiteracy. As a result, we decided to study the subjects by a face-to-face structured interview rather than a paper-pencil survey. Second, in the formal study, four questions were asked as the inclusion criteria: Where are you from? Do you possess the rural hukou or urban hukou? Are you unemployed? Do you lost your job at least one month? Only subjects who met these criteria were asked to complete the study. Third, the interview was conducted in the quiet and comfortable office rooms in the labour markets because it provided adequate privacy and was convenient to the participants. Before each interview, the eligible participant was explained about the purpose and the procedure of the study and asked for their consent. The individual-based face-to-face interview took approximately 30 minutes and was conducted by trained researchers, including faculty members and postgraduate students from Wenzhou Medical College. Systematic training was provided before these researchers were dispatched to the field to conduct the interviews. Fourth, upon completion, each of participants was given a tooth brush and a tooth paste as a token of appreciation.

This study was done in compliance with the Helsinki Declaration, and was reviewed and approved by the Ethics Committee of Wenzhou Medical College.

### Data analysis

First, we created dichotomous variables (unhealthy vs. healthy) for each subscale of SCL-90-R, considering subscale scores > 2 or total scores of global severity index (GSI) > 70 as suggestive of possible psychopathology. Second, independent sample t tests were conducted with each SCL-90-R subscale between the unemployed migrant worker samples and the Chinese adult norm of 1986 [42], 2003 [45] and 2006 [42]. Third, we compared scores of SCL-90-R sub-scales among long-term, short-term unemployed migrant workers and employed migrant workers by two One-Way Analysis of Variance (ANOVA). The data source of employed migrant workers came from the research of Liao’s group, in which 345 employed migrant workers in Wenzhou were measured by SCL-90-R during the economic crisis of 2008 [46]. Fourth, two ANOVA also were carried out on each SCL-90-R subscale among three groups by types of coping strategy. Finally, hierarchical regression analysis was used to measure the effects of the related factors on mental health.

## Results

### Socio-demographics of the unemployed migrant workers

Of the 210 respondents, 135 (64.3%) were males and 75(35.7%) females, 116(55%) of unemployed migrant workers were younger than 30, and over one-third of them were single. Most of the unemployed migrants only had a junior secondary school education (5-8 years of schooling) or below, and 138 (65.7%) of them were short-term unemployed migrant workers. About 86.7% of these migrant workers worked as manufacturing industry workers and 7.6% as service industry workers. The prevalence of main social and demographic characteristics by type of unemployment is presented in Table 1. It demonstrates that the majority of the unemployed migrant workers were young male manufacturing industry workers with short-term unemployment and a relatively low education level.

**Table 1:** **Socio-demographics of the unemployed migrant workers (*N*** **= 210)**

**Variables**		***n***	***%***
Gender	Men	135	64.3
	Women	75	35.7
Age groups	<20	25	11.9
	21-30	91	43.3
	31-40	60	28.6
	>41	34	16.2
Marital status	Single	72	34.3
	Married/Cohabiting	122	58.1
	Divorced/Widowed	16	7.6
Education	<5 years or illiteracy	24	11.4
	5-8 years	98	46.7
	9-11 years	62	29.5
	>11 years	26	12.4
Type of last job	Shoe industry	91	43.3
	Grasses industry	48	22.9
	Closing industry	41	19.5
	Mechanical industry	2	1.0
	Catering	16	7.6
	Others	12	5.7
region of origin	Jiangxi province	48	22.8
	Anhui province	36	17.1
	Hunan province	39	18.6
	Henan province	24	11.4
	Sichuan province	25	11.9
	Other provinces	38	18.1
Duration of unemployment	≤3 months	138	65.7
	>3 months	72	34.3

### Mental health of the unemployed migrant workers

**Figure 1:**
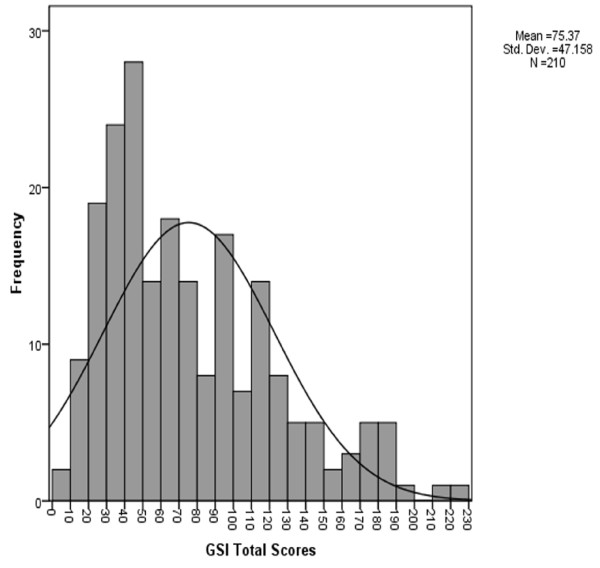
**Distribution of GSI total scores.** Histogram showing the distribution of the Symptom Checklist-90-Revised (SCL-90-R) global severity index (GSI) total scores in the unemployed migrant workers. GSI total scores > 70 identify cases with possible mental disorder (SCL cases: *n* = 94 out of 210 subjects).The y-axis shows the number of individuals with a GSI score in the ranges of the GSI indicated in the x-axis (note that ten GSI points are condensed into one category on the x-axis).For example, 26 subjects had a GSI score in the range of 60-70.The range of the observed scores was 0-230, Mean = 75.37, SD = 47.16.

In general, as shown in Figure 1, the mean SCL-90-R GSI scores for all 210 unemployed migrant workers was above the cut-off point (GSI > 70), with a positive skewed distribution, 94 (45%) had a GSI > 70, indicating marked psychological distress and probable psychological/psychiatric illness.

Specifically, according to the criteria (subscale scores > 2 as suggestive of possible psychopathology), 107(51%) had a DEP > 2, 102(49%) had a PAR > 2, 101 (48%) had a HOS > 2, 91 (43%) had a INS > 2, 84 (40%) had a O-C > 2, 77 (37%) had a ANX > 2, 70 (33%) had a SOM > 2, 53 (25%) had a PSY > 2, and 42 (20%) had a PHOB > 2.

To determine whether the unemployed migrant workers would report elevated levels of distress compared with the adult norm in China, independent t-tests were conducted with each SCL-90-R subscale. As shown in Table 2, on all nine clinical subscales of SCL-90-R, unemployed migrant workers scored significantly higher than the adult norms of 1986 [42], 2003 [45] and 2006 [42].

**Table 2:** Comparison of scores of SCL-90-R sub-scales between the unemployed migrant workers and the adult norm

		**Scores of SCL-90-R sub-scales (M ± SD)**								
		**SOM**	**O-C**	**INS**	**DEP**	**ANX**	**HOS**	**PHOB**	**PAR**	**PSY**
Samples of unemployed migrant workers	(*N* = 210 )	1.73 ± 0.59	1.95 ± 0.64	1.92 ± 0.55	2.02 ± 0.04	1.81 ± 0.58	1.97 ± 0.74	1.56 ± 0.59	1.98 ± 0.58	1.64 ± 0.55
The adult norm of 1986^*a*^	(*N* = 1388)	1.37 ± 0.48	1.62 ± 0.58	1.65 ± 0.61	1.50 ± 0.59	1.39 ± 0.43	1.46 ± 0.55	1.23 ± 0.41	1.43 ± 0.57	1.29 ± 0.42
*t*^*1*^		1.88^***^	7.44^***^	7.28^***^	12.35^***^	10.61^***^	9.97^***^	8.15^***^	13.91^***^	9.16^***^
The adult norm of 2003^b^	(*N* = 2808 )	1.36 ± 0.39	1.47 ± 0.45	1.44 ± 0.45	1.33 ± 0.39	1.30 ± 0.37	1.36 ± 0.41	1.17 ± 0.30	1.32 ± 0.42	1.25 ± 0.34
*t*^*2*^		8.99^***^	10.84^***^	12.86^***^	16.37^***^	12.88^***^	11.94^***^	9.61^***^	16.67^***^	10.22^***^
The adult norm of 2006^c^	(*N* = 1890 )	1.42 ± 0.44	1.66 ± 0.52	1.51 ± 0.49	1.49 ± 0.47	1.34 ± 0.39	1.49 ± 0.51	1.27 ± 0.39	1.44 ± 0.47	1.33 ± 0.39
*t*^*3*^		7.54^***^	6.53^***^	10.99^***^	12.59^***^	11.87^***^	9.38^***^	7.16^***^	13.66^***^	8.112^***^

Note: ^*^p < 0.05 ^**^p < 0.01 ^***^p < 0.001.

^1^Comparison of Scores of SCL-90-R Sub-scales between the unemployed migrant workers and the adult norm of 1986.

^2^Comparison of Scores of SCL-90-R Sub-scales between the unemployed migrant workers and the adult norm of 2003.

^3^Comparison of Scores of SCL-90-R Sub-scales between the unemployed migrant workers and the adult norm of 2006.

^a, c^For the data source, see reference No.42.

^b^For the data source, see reference No.45.

*SOM* Somatization, *INS* Interpersonal Sensitivity, *ANX* AnxietyPHOB: Phobic Anxiety, *PSY* Psychoticism.

*O-C* Obsessive-Compulsive, *DEP* Depression, HOS Hostility, *PAR* Paranoid Ideation, *SCL-90-R* Symptom Checklist-90-Revised.

### Mental health and duration of unemployment

**Figure 2:**
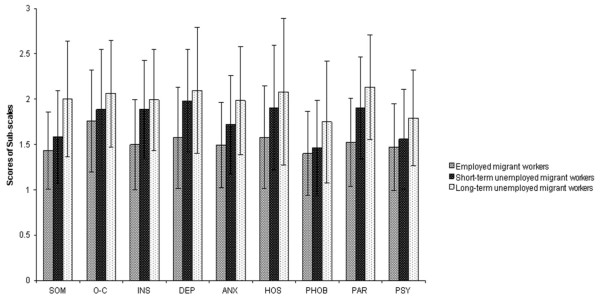
**Symptom profile (SCL-90-R sub-scales) for employed, short-term unemployed and long-term unemployed migrant workers.** SCL-90-R: Symptom Checklist-90-Revised; SOM: Somatization; O-C: Obsessive-Compulsive; I-S: Interpersonal Sensitivity; DEP: Depression; ANX: Anxiety; HOS: Hostility; PHOB: Phobic Anxiety; PAR: Paranoid Ideation; PSY: Psychoticism.

The correlation matrix presented in Table 3 revealed that duration of unemployment has significant positive correlations with anxiety(r = 0.22, *p* < 0.01), paranoid ideation(r = 0.19, *p* < 0.01), psychoticism(r = 0.20, *p* < 0.01). As shown in Table 4, unemployed migrant workers expressed significantly more symptoms of psychological distress in nine SCL-90-R subscales, whether it is short-term or long-term unemployment. Long-term unemployed migrant workers expressed significantly more symptoms of psychological distress than short-term unemployed (somatization, anxiety, phobic anxiety, paranoid ideation, psychoticism). To further illustrate Table 4, Figure 2 depicts the SCL-90-R profile for long-term unemployed, short-term unemployed and employed migrant workers.

**Table 3:** **Intercorrelations among variables used in this study (*N*** **= 210)**

	**1**	**2**	**3**	**4**	**5**	**6**	**7**	**8**	**9**	**10**	**11**	**12**	**13**	**14**	**15**	**16**	**17**	**18**	**19**	**20**
1.Gender	-																			
2.Age	-.09	-																		
3.Marital status	-.20**	.67**	-																	
4.Education	.06	.00	.02	-																
5.Problem-solving	-.19**	.22**	.10	.12	-															
6.Self-blame	-.05	-.21**	-.06	-.05	-.33**	-														
7.Help-seeking	-.14*	-.01	.04	-.03	.23**	-.02	-													
8.Fantasizing	-.06	-.12	.06	.02	-.25**	.62**	-.03	-												
9.Avoidance	-.02	.11	.12	.03	.19**	.30**	.07	.31**	-											
10.Rationalization	-.12	-.12	.06	.08	-.11	.66**	.10	.66**	.40**	-										
11.Duration unemployment	-.06	.06	.06	.11	-.29**	.03	-.12	.10	-.11	.01	-									
12.SOM	.06	-.07	-.01	-.13	-.49**	.35**	.04	.31**	-.12	.19**	.34**	-								
13.O-C	.11	-.21**	-.25**	-01*	-.45**	.44**	-.01	.29**	.04	.17*	.13	.66**	-							
14.INS	.06	-.19**	-.16*	-.13	-.32**	.55**	-.03	.38**	.16*	.29**	.09	.59**	.77**	-						
15.DEP	.02	-.07	-.07	-.09	-.31**	.57**	-.09	.32**	.19**	.29**	.09	.65**	.78**	.80**	-					
16.ANX	.13	-.11	-.07	-.07	-.47**	.42**	-.03	.35**	-.02	.22**	.22**	.82**	.79**	.75**	.79**	-				
17.HOS	.23**	-.25**	-.15*	-.02	-.39**	.46**	.12	.36**	.20**	.40**	.12	.62**	.65**	.67**	.70**	.72**	-			
18.PHOB	.11	-.05	-.06	-.10	-.53**	.46**	-.06	.36**	-.09	.21**	.23**	.78**	.71**	.75**	.69**	.78**	.58**	-		
19.PAR	.19**	-.14*	-.11	-.02	-.39**	.53**	-.04	.41**	.16*	.34**	.19**	.67**	.74**	.79**	.82**	.77**	.76**	.74**	-	
20.PSY	.22**	-.17*	-.13	-.12	-.49**	.51**	.01	.34**	-.08	.24**	.20**	.79**	.77**	.74**	.68**	.83**	.65**	.82**	.78**	-

Note: ^*^p < 0.05 ^**^p < 0.01 ^***^p < 0.001;

*SOM* Somatization, *INS* Interpersonal Sensitivity, *ANX* Anxiety, *PHOB* Phobic Anxiety, *PSY* Psychoticism.

*O-C* Obsessive-Compulsive, *DEP* Depression, *HOS* Hostility, *PAR* Paranoid Ideation.

**Table 4:** Comparison of Scores of SCL-90-R sub-scales among long-term, short-term unemployed migrant workers and employed migrant workers

**SCL-90-R sub-scales**	**M ± SD**			***F***	**Mean difference between long-term unemployed and short-term unemployed**	**Mean difference between long-term unemployed and employed**	**Mean difference between short-term unemployed and employed**
	**Long-term unemployed migrant workers(*n*** **= 73)**	**Short-term unemployed migrant workers (*n*** **= 137)**	**Employed migrant workers**^**a**^**(*n*** **= 347)**				
SOM	2.00 ± 0.64	1.58 ± 0.51	1.43 ± 0.42	43.68***	0.42***	0.57***	0.15**
O-C	2.06 ± 0.59	1.89 ± 0.66	1.76 ± 0.56	8.97***	0.17*	0.31***	0.13*
INS	1.99 ± 0.56	1.89 ± 0.54	1.49 ± 0.49	45.69***	0.10	0.49***	0.39***
DEP	2.10 ± 0.69	1.98 ± 0.56	1.57 ± 0.56	40.10***	0.11	0.52***	0.41***
ANX	1.98 ± 0.59	1.72 ± 0.54	1.49 ± 0.47	32.31***	0.26***	0.49***	0.23***
HOS	2.08 ± 0.81	1.90 ± 0.69	1.57 ± 0.57	26.21***	0.18	0.50***	0.33***
PHOB	1.75 ± 0.67	1.46 ± 0.52	1.40 ± 0.46	14.00**	0.28***	0.37***	0.06
PAR	2.13 ± 0.58	1.90 ± 0.56	1.52 ± 0.49	56.67***	0.23**	0.61***	0.38***
PSY	1.79 ± 0.53	1.56 ± 0.55	1.47 ± 0.48	12.55***	0.24**	0.32***	0.09

Note:^*^p < 0.05 ^**^p < 0.01 ^***^p < 0.001.

^a^ For the data source, see reference No.46.

*SOM* Somatization, *INS* Interpersonal Sensitivity, *ANX* Anxiety, *PHOB* Phobic Anxiety, *PSY* Psychoticism.

*O-C* Obsessive-Compulsive, *DEP* Depression, *HOS* Hostility, *PAR* Paranoid Ideation, *SCL-90-R* Symptom Checklist-90-Revised.

### Mental health and coping strategy of unemployed migrant workers

The most frequently used coping strategies by the unemployed migrant workers were problem-solving (0.73 ± 0.20), followed by avoidance (0.62 ±0.16), rationalization (0.61 ± 0.17), fantasizing (0.56 ± 0.21), help-seeking (0.55 ± 0.19) and self-blaming (0.48 ± 0.28).

**Figure 3:**
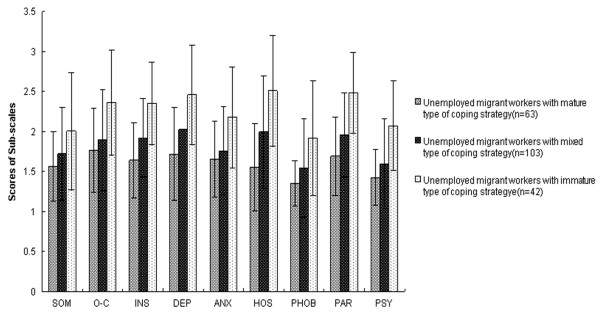
**Symptom profile (SCL-90-R sub-scales) for unemployed migrant workers with three types of coping strategy.** Figure 3 presented the three groups of unemployed migrant workers (shaded bars): unemployed migrant workers with mature type of coping strategy, unemployed migrant workers with mixed type of coping strategy, and unemployed migrant workers with immature type of coping strategy. SCL-90-R: Symptom Checklist-90-Revised; SOM: Somatization; O-C: Obsessive-Compulsive; I-S: Interpersonal Sensitivity; DEP: Depression; ANX: Anxiety; HOS: Hostility; PHOB: Phobic Anxiety; PAR: Paranoid Ideation; PSY: Psychoticism.

The correlation matrix presented in Table 3 revealed that there are significant associations between the coping strategies and mental health symptoms of unemployed migrant workers. As shown in Table 5, unemployed migrant workers with immature type of coping strategy expressed significantly more symptoms of psychological distress compared to unemployed migrant workers with mixed or mature type of coping strategy in nine SCL-90-R subscales. Unemployed migrant workers with mixed type of coping strategy also expressed a more pronounced symptom load when compared to mature type group in interpersonal sensitivity, depression, hostility, paranoid ideation, and psychoticism. To further illustrate Table 5, Figure 3 depicts the profile of SCL-90-R scores for different types of coping strategy.

**Table 5:** Comparison of Scores of SCL-90-R sub-scales among unemployed migrant workers with three types of coping strategy

**SCL-90-R sub-scales**	**Type of coping strategy (M ± SD)**			***F***	**Mean difference between mature type and mixed type**	**Mean difference between mature type and immature type**	**Mean difference between mixed type and immature type**
	**Mature type (*n*** **= 63)**	**Mixed type (*n*** **= 103)**	**Immature type (*n*** **= 42)**				
SOM	1.57 ± 0.43	1.72 ± 0.58	2.01 ± 0.73	7.30**	-0.15	-0.44***	-0.28**
O-C	1.76 ± 0.52	1.89 ± 0.63	2.36 ± 0.65	13.16***	-0.13	-0.59***	-0.05***
INS	1.64 ± 0.47	1.92 ± 0.49	2.35 ± 0.51	26.38***	-0.28***	-0.71***	0.43***
DEP	1.72 ± 0.58	2.03 ± 0.52	2.46 ± 0.62	21.94***	-0.37**	-0.74**	-0.43***
ANX	1.66 ± 0.48	1.76 ± 0.55	2.18 ± 0.63	12.37***	-0.10	-0.52***	-0.42***
HOS	1.55 ± 0.55	1.99 ± 0.71	2.51 ± 0.69	26.85***	-0.44***	-0.96***	-0.52**
PHOB	1.35 ± 0.28	1.54 ± 0.62	1.92 ± 0.72	12.76***	-0.19*	-0.56***	-0.37***
PAR	1.69 ± 0.49	1.97 ± 0.52	2.48 ± 0.51	30.83***	-0.27***	-0.79**	-0.53***
PSY	1.42 ± 0.35	1.59 ± 0.56	2.07 ± 0.56	21.58***	-0.17***	-0.65***	-0.48***

Note: ^*^p < 0.05 ^**^p < 0.01 ^***^p < 0.001.

*SOM* Somatization, *INS* Interpersonal Sensitivity, *ANX* Anxiety, *PHOB* Phobic Anxiety, *PSY* Psychoticism.

*O-C* Obsessive-Compulsive, *DEP* Depression, *HOS* Hostility, *PAR* Paranoid Ideation, *SCL-90-R* Symptom Checklist-90-Revised.

### Hierarchical regression analysis for predicting GSI

As shown in Table 6, duration of unemployment and two coping strategies, problem-solving and self-blaming, significantly predicted the mental health of the unemployed migrant workers. Duration of unemployment contributed to about 5% of the variance in total scores of GSI and problem-solving and self-blaming explained over 33% of the variance in total scores of GSI.

**Table 6:** **Results of hierarchical regression analysis (*N*** **= 210)**

**Independent variables**		**GSI total score**			
		**Step1**	**Step2**	**Step3**	
				**B (SE)**	***β***^***a***^
1. Control variables	Gender			11.57^*^(5.64)	0.12
	Age			4.30 (3.98)	0.08
	Marital status			-11.17 (7.41)	-0.11
	Education(years)			-3.72 (3.07)	-0.07
2. Duration unemployment(months)				13.11 (5.69)	0.13^*^
3. Coping strategy	Problem-solving			-63.98 (15.97)	-0.27^***^
	Self-blaming			74.72 (13.39)	0.45^***^
	Help-seeking			24.89 (13.45)	0.10
	Fantasizing			25.69 (17.14)	0.12
	Avoidance			-7.57 (18.30)	-0.03
	Rationalization			-22.13 (23.01)	-0.08
*R*^*2*^		0.05	0.11	0.45	
*Adjust R*^*2*^		0.04	0.09	0.42	
*F*		2.93^*^	5.16^***^	14.42^***^	
Δ*R*^*2*^			0.06	0.33	
Δ*F*			13.36^***^	19.75^***^	

Note: ^*^p < 0.05 ^**^p < 0.01 ^***^p < 0.001.

^a^standardized regression coefficients at each step.

## Discussion

### Limitations of the study

Four limitations are present prior to proceeding with the discussion on the results of this study. First, we used a convenience sampling method, which may limit the generalizablity of the findings to the target population. Second, data were self-reported in nature and may be subject to reporting bias, although most mental health studies are in fact self-reported. Third, this study did not collect the job characteristics (i.e., duration of last job) that may have an association with mental health status during unemployment, an important topic for future studies. Fourth, this study is not longitudinal. The characteristic of migrant workers did not permit follow-ups of the participants during their full unemployment period for an evaluation of their mental health.

### Mental health of the unemployed migrant workers

This study is the first estimation of prevalence of mental problems among unemployed migrant workers in Eastern China. It is found that as high as 50 percent of the unemployed migrant workers are classified as mentally unhealthy. Compared with the SCL-90-R adult norm of 1986, 2003, and 2006 in China, the unemployed migrant workers exhibited much poorer mental health and the most frequently reported item was depression. The prevalence rate found in the study is much higher than that reported in previous studies [23,24]. For instance, in the study conducted by Wong et al, 31% migrant workers in China were classified as mentally unhealthy [23].

Both unemployment status and migration status might explain the high prevalence rate of mental problems among unemployed migrant workers in Eastern China. First, during the economic crisis, as the existing literature has indicated [6,8], unemployed individuals have witnessed the loss of property and jobs as well as social disruption and increasing deterioration of quality of life and well-being. These adverse changes were significantly associated with mental health problems, particularly with depression. Second, migration stress entails tremendous social and economic cost but with uncertain benefit. It has often been seen as a potential risk factor that can increase the likelihood of poor mental health outcomes [47]. In China, because of the *hukou* system, migrant workers cannot register as formal residents in cities and therefore are not entitled to subsidized housing, education, social security, or medical benefits. Studies also suggest that they tend to live in poorly sanitized and usually overcrowded dormitories [23,24]. Thus, unemployed migrant workers in China have to face double pressures due to their unemployment and migration status, leading to a high rate of mental problems.

### Mental health and duration of unemployment

There are two major findings in the study relevant to mental health and duration of unemployment in this study. First, in line with the earlier findings in Western countries [4,10,27], our study found that unemployed migrant workers expressed significantly more symptoms of psychological distress, compared with the employed migrant workers in China. Second, the mental problems of long-term unemployed migrant workers were more serious than that of short-term ones. These results are consistent with some of the previous research into long-term unemployment [26-28], but not with other research indicating that the job loss is the primary contributor and intensified most at the time it occurs, subsiding later [29].

There are possible explanations for why long-term unemployed migrant workers in Eastern China have more mental problems. As the existing literature has indicated, being without a job for a long period of time allows stress to accumulate, coping resources to be depleted, and anxiety and tension to mount due to unemployment benefits running out and savings being exhausted [48,49]. Thus, the financial detriment of job loss increases as unemployment duration extends [10,26,27]. As a result, individuals with longer unemployment duration in China did show lower levels of mental health. On the other hand, for those short-term unemployed migrant workers in Eastern China, their initial challenging experience of migrating from rural villages to urban cities have equipped them with important life lessons and basic survival skills. Thus, their subsequent daily experience of obtaining and losing jobs temporarily might not become the sole or largest stressor in their lives. They still have their dreams for their future and might even have savings left. As a result, short term unemployed migrant workers showed less mental problems that those long term ones.

### Mental health and coping strategy of unemployed migrant workers

The most commonly used coping strategy for unemployed migrant workers is problem-solving, indicating that they, in the face of difficulty and pressure, would take some positive and mature coping strategies to coping with it. This result is consistent with some employed worker research [50,51], but inconsistent with other unemployed worker research indicating that problem-solving was less frequent used among unemployed subjects [52].

Self-blaming and help-seeking rank the least, which are congruent with the previous research into coping strategies used by migrant workers in China [21]. It is possible that individuals in the sample would attribute the cause of their unemployment to the poor leadership of their unit and the inevitable result of the enterprise bankrupt during the economic crisis, and consider it little to do with individual abilities and qualities. Interestingly, findings from this study indicate that unemployed migrant workers use the coping strategy of help-seeking less. Unemployed migrants felt they needed help when they were depressed or anxious more than either urban or rural counterparts [21]. However, they were least likely to actually take action to get help. Unemployed migrant workers are separated from their accustomed support network, such as family or friends in the rural areas. Meanwhile, it is impossible for them to get the help from professional counselling and psychological support services due to its limitation and costliness in China [53].

With regards to the relationship between mental health status and the coping strategies utilized by unemployed migrant workers, mature coping strategy (e.g. problem-solving) and mixed coping strategy (e.g. rationalization) showed significant negative correlation with all the factors of SCL-90-R. In contrast, immature coping strategy (e.g. self-blaming, fantasizing) showed significant positive correlation with the factors. This finding is consistent with previous coping studies [54-56]. According to the research of Lazarus and Folkman, individuals with mature coping strategy could make direct efforts to alter or manage the source of the problem, which resulted in reducing anxiety and improving mental health [44]. In contrast, immature coping strategies are associated with psychological distress [57], high level of depression [58,59] and negative affect [60-62]. Although immature coping strategies can temporarily be used to escape the threat of pressure, yet the pressures are still there and have to be faced sooner or later, so they will lower the level of mental health. Comparison of SCL-90-R scores for different types of coping strategy also indicate that those who usually adopted mature type had comparatively good mental health; those who commonly used immature type had poor mental health; and those who were most likely to use mixed type between the above two types (mature and immature) were moderately mentally healthy.

### Hierarchical regression analysis for predicting GSI

The regression analysis further revealed that duration of unemployment and two kinds of coping strategies (problem-solving and self-blaming) were the primary predictors of the mental health of unemployed migrant workers in our study. This finding might have practical implications. To prevent and address mental problems among unemployed migrant workers in China, for example, study on predicted factors may help policymakers develop programs to reduce the length time of unemployment and teach them how to increase the use of the problem-solving strategies and decrease the use of the self-blaming strategies.

## Conclusions

This study indicates the mental problem of unemployed migrant workers was much more serious and widespread in Eastern China during the economic crisis. Duration of unemployment and two coping strategies, problem-solving and self-blaming are significant factors influencing their mental health. Given the massive number of unemployed migrants in China, their mental health should receive more attention. Despite limited resources, mental health could be improved through not only health care services but also social reforms. It is time for policymakers to develop new initiatives and to consolidate the existing policies that have been implemented in order to protect the mental health of the millions of unemployment migrant workers in China. It is important to note that the present study, using the cross-sectional design, has confirmed that unemployment and poor mental health are significantly related in one direction: unemployment may worsen mental health. However, the relationship could also work in reverse directions: mental health problems, particularly the common mental disorders such as anxiety and depression, may make it more difficult for a person to obtain and/or hold a job. Therefore, the reverse causal direction should be further examined in the future longitudinal studies.

## Abbreviations

CSQ: Coping Styles Questionnaire;SCL-90-R: Symptom Check List 90;SOM: Somatization;O-C: Obsessive-Compulsive;I-S: Interpersonal Sensitivity;DEP: Depression;ANX: Anxiety;HOS: Hostility;PHOB: Phobic Anxiety;PAR: Paranoid Ideation;PSY: Psychoticism;GSI: Global Severity Index.

## Competing interests

The authors declare that they have no competing interests.

## Authors’ contributions

CL designed the study, conducted the data analysis, and completed the first draft of this article. WJT participated in study design and coordination. LHW, WHL and JCH participated in the design of the study, and revised the manuscript. YZ made valuable suggestions on scholarly writing. All authors have read and approved the final manuscript.

## Pre-publication history

The pre-publication history for this paper can be accessed here:

http://www.biomedcentral.com/1471-2458/12/597/prepub
